# Coexistence of p190 *BCR/ABL* Transcript and *CALR* 52-bp Deletion in Chronic Myeloid Leukemia Blast Crisis: A Case Report

**DOI:** 10.4084/MJHID.2016.002

**Published:** 2016-01-01

**Authors:** Mohammad Seghatoleslami, Neda Ketabchi, Alireza Ordo, Javad Mohammadi Asl, Neda Golchin, Najmaldin Saki

**Affiliations:** 1 Health Research Institute, Research Center of Thalassemia and Hemoglobinopathy, Ahvaz Jundishapur University of Medical Sciences, Ahvaz, Iran; 2 Department of Medical Genetics, Faculty of Medicine, Ahvaz Jundishapur University of Medical Sciences, Ahvaz, Iran

## Abstract

We introduce a 78-year-old woman presented with thrombocytosis and high blast count who had a history of splenectomy. Her cytogenetic analysis revealed aberrant chromosomal rearrangements in different clonal populations harboring 46XX karyotype with t(9;22) (q34;q11). RT-PCR assay detected the e1a2 *BCR-ABL* translocation resulting from rearrangement of the minor breakpoint cluster region (m-bcr) in *BCR* gene. Subsequent evaluation of the disease showed calreticulin (*CALR*) 52-bp deletion as well as the absence of *JAK2*^V617F^ heterozygous mutation in granulocyte population of peripheral blood using allele-specific PCR and bi-directional DNA sequencing. To our knowledge, this is the first case of a patient initially diagnosed as p190 *BCR-ABL* transcript positive CML in blast crisis characterized by a 52-bp deletion in *CALR* gene.

## Introduction

Chronic myeloid leukemia (CML) is the most important myeloproliferative neoplasm (MPN) developed due to the known t(9;22)(q34;q11) chromosomal translocation in pluripotential hematopoietic progenitor cells. This genetic alteration results in the formation of Philadelphia chromosome (Ph′), comprising portions of Abelson (*abl*) and breakpoint cluster region (*bcr*) genes.^1^,^2^ This fusion transcript is present in 90–95% of patients with CML andserves as adiagnostic and prognostic biomarker. It is also a therapeutic target in cases with a wide spectrum of clinical symptoms, including hypercellular bone marrow (BM), splenomegaly, anemia or platelet dysfunction as well as significant increase in the number of leukocytes, especially neutrophils and immature myeloid cells.^3^ Unlike the common form of p210 BCR-ABL oncoprotein, in which the breakpoint occurs between exon 2 of *abl*gene on chromosome 9 and exons e12–e16 of the *bcr*gene on chromosome 22, the first exon of *bcr*gene is involved in this translocationin 1–2% of CML patients. This rare fusion transcript, also known as minor breakpoint cluster region (m-bcr), codes for a smaller (190kDa) oncoprotein with a unique clinical presentation between CML and chronic myelomonocytic leukemia (CMML).^4^

Besides the identification of mutations in Janus kinase 2 (*JAK2*) and thrombopoietin receptor (*MPL)* genes BCR/ABL negative MPNs, calreticulin (*CALR*) gene mutations have also been used in classification and determination of diagnostic criteria for MPNs and myelodysplastic/myeloproliferative neoplasms (MDS/MPN), including essential thrombocythemia (ET), primary myelofibrosis (PMF) and refractory anemia with ring sideroblasts associated with marked thrombocytosis (RARS-T), respectively.^5^ Insertion/deletion mutations in exon 9 of *CALR* does not occur in CML, but some authors have recently reported the existence of these somatic changes in remaining portions of nonmutated *JAK2* and *MPL* MPN cases.^6^

We report the case of a CML patient with p190 type *BCR*–*ABL* transcript who also harbored *CALR* 52-bp deletion. Beyond a few studies of CML patients with p190 kDa fusion protein,^7^ to the best of our knowledge, this is the first report to describe the coexistence of P190 *BCR/ABL* transcript and *CALR* 52-bp deletion in blast crisis in a CML patient. Herewith, we have presented a detailed insight into the study of clinical and molecular cytogenetic findings to assess the prognostic information in guiding management strategies for our patient.

## Case report

**Figure 1:**
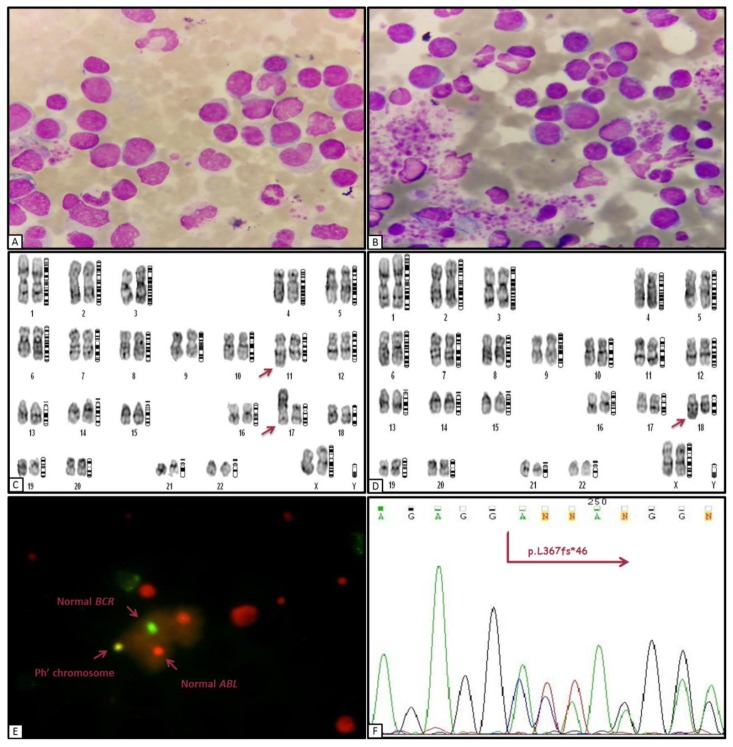
**A, B:** Increased blasts and platelets in BM examination of initial diagnosis. **C, D:** Chromosomal aberrations revealed in cytogenetic analysis. **E:** FISH indicated the rearrangement between ABL and BCR genes. **F:** Bi-directional sequencing for confirmation of CALR 52-bp mutation.

A 78-year-old woman was admitted to our department in May 2015 with pallor, weakness and a remote history of splenectomy. Her peripheral blood (PB) revealed anemia with a hemoglobin concentration of 8.6 g/dL, thrombocytosis (789,000×10^3^/μL), white blood cell (WBC) count of 68200/μL with 16% neutrophils, 1% eosinophils, 1% monocytes, 48% lymphocytes, 16% atypical lymphocytes and 18% blasts. Morphologic review of the PB smear revealed basket cells and nucleated red blood cells. BM aspirate smears showed hypercellular marrow with increased blasts (Figure 1A) and platelets (Figure 1B). Blasts showed a fine chromatin pattern, round nuclei, and scanty cytoplasm.

Cytogenetic analysis indicated the presence of der(11), der(17) and der(18) chromosome abnormalities in different clonal populations harboring 46XX karyotype with t(9;22) (q34;q11) (Figure 1C, 1D) in addition to the observed *BCR-ABL* fusion gene in BM metaphases by fluorescence in situ hybridization (FISH) (Figure 1E). In the first step of verification at the molecular level, RT-PCR was performed for detection of p210-type mRNA as previously described,^8^ but sequence analysis of amplification products did not show the p210 *BCR-ABL* positive rearrangement. This result prompted us to investigate p190*BCR-ABL* mRNA fusion transcript by RT-PCR assay according to standard procedures, which was positive in our patient similar to rare cases of CML with an inferior outcome of therapy.^9^

The initial manifestation of the disease was an overwhelming splenomegaly; however, the patient was referred to our center with a high platelet count anterior to splenectomy. In consideration of the possibility of a chronic myeloproliferative disease transformed in the acute phase, it has been carried out allele-specific PCR to detect the mutation JAK2V617F. Combining the previously published studies with current data, including a high platelet count and the absence of *JAK2*^V617F^ mutation, we were encouraged to study mutations in exon 9 of *CALR* by bidirectional sequencing in the following investigations (Figure 1F),^10^ which surprisingly revealed a del52CALR mutant with a high allele burden in granulocyte population.

## Discussion

We report the case of an untreated CML patient bearing p190 *BCR/ABL* transcript and *CALR* 52-bp deletion with additional chromosomal aberrations. The patient had a dramatic thrombocytosis as well as high WBC count. Although rarely reported, exclusive expression of e1a2BCR-ABL translocation is associated with highly divergent clinical outcomes. Previous studies suggest no relationship between distinct clinical presentations of CML and type of BCR-ABL rearrangement^11^ while many others put them in a high-risk category at diagnosis with an early transformation to blast phase similar to our patient.^12^,^13^

CMML-like phenotype with monocytosis seems to be a useful diagnostic picture for most cases of p190 BCR-ABL CML especially in chronic phase,^11^ but we advocate the consideration of more detailed analysis to prevent any delay in therapeutic interventions for p190 blast crisis cases due to lack of distinct clinical and biological features. Xu et al. reviewed 17 cases of CML patients expressing p190 BCR-ABL oncoprotein and *JAK2*^V617F^ mutations who almost achieved a good response during treatment with tyrosine kinase inhibitors (TKIs).^14^ This issue raises many questions about the presence of *CALR* mutation in p190 BCR-ABL CML and its likely impact on the clinical course and prognosis of our patient. So far, *CALR* mutation status has been associated with thrombocytosis in MPNs, including ET and PMF.^10^ We believe that the presence of *CALR* 52-bp deletion in our CML case imparts the high platelet count and ultimately mimics its progression toward cases other than CML in MPNs. Since platelet count acts as a prognostic factor in the evaluation of response to TKIs, we suggest the detection of *CALR* mutation in all p190 BCR-ABL CML patients initially presenting with thrombocytosis given the prognosis and treatment strategies in such cases.^15^ In summary, this interesting case illustrates that *CALR* 52-bp deletion may act as a distinctive feature in the diagnosis of p190 BCR-ABLCML patients. Questions remain regarding the exact contribution of *CALR* deletion to MPNs.
